# Interactions Between HEP Peptide and EGFR Involved in the Osteoblast Differentiation

**DOI:** 10.3390/foods14173032

**Published:** 2025-08-29

**Authors:** Jing Gan, Yanling Huang, Mengqi Jian, Yuhang Chen, Yuxuan Jiang, Yang Qiao, Yang Li

**Affiliations:** 1 Heilongjiang Green Food Science Research Institute, Harbin 150028, China; ganjing@ytu.edu.cn (J.G.); cinderella32@163.com (Y.H.); 2 College of Life Science, Yantai University, Yantai 264000, China; jianmengqi1999@163.com (M.J.); cyhzzzz@163.com (Y.C.); 17325483605@163.com (Y.J.); qy09156015@163.com (Y.Q.); 3 Beijing Key Laboratory of Functional Food from Plant Resources, College of Food Science and Nutritional Engineering, China Agricultural University, Beijing 100083, China

**Keywords:** peptide, epidermal growth factor receptor, binding mechanism, molecular docking analyses, spectroscopic methods

## Abstract

The epidermal growth factor receptor (EGFR), as an important target protein for inhibiting and intervening in osteoporosis, is associated with cell migration, proliferation, and apoptosis. Peptides derived from food have been shown to have a strong affinity for EGFR, thereby regulating downstream cellular-signaling pathways and participating in stimulating bone formation. However, it is still a “black box” as to how active peptides affect the conformational changes in the EGFR-binding domain when interacting with its ligand EGF. To shed light on the roles, peptides in EGFR binding, which is involved in the osteoblast differentiation, a high EGFR affinity soybean peptide (HEP) was isolated and purified from soy yogurt. Firstly, the osteogenic activity of HEP was identified through cellular alkaline-phosphatase (ALP) and calcium influx. HEP promoted ALP activity from 0.01897 ± 0.00165 to 0.04051 ± 0.00402 U/mg after 100 μM of peptide treatment, and free intracellular calcium ions and calcium deposition both increased in a dose-dependent manner at 1–100 μg/mL. Secondly, the interaction between HEP and EGFR was detected by bioinformatics, spectroscopy analysis, and Western blot. The Molecular docking results showed that HEP (VVELLKAFEEKF) exhibited high affinity among all the peptides, with -CDOCKER energy values of 184.077 kcal/mol on one EGFR. Moreover, a different loop conformation has been detected in HEP, comparing it to that of EGF, which influences HEP interactions with EGFR. GlU3, LEU4, and LEU5 (HEP) match GLU40, LEU26, and GLU40 (EGF). Moreover, the CD data showed that HEP could interact with extracellular domain protein of EGFR, but the secondary structure did not change after HEP was mixed with Mutant extracellular domain protein. Furthermore, treatment with HEP increased the expression of EGFR and the activation of the PI3K-RUNX2-signaling pathway. These results suggested that HEP may have the function of promoting bone remodeling, which could promote the binding between EGF and EGFR and may be used as a potential active factor for functional food development to prevent osteoporosis.

## 1. Introduction

Osteoporosis, a prevalent metabolic bone disorder affecting millions globally, ranks as the fifth-most-common disease. It is marked by decreased bone mineral density (BMD) and heightened bone fragility. Bone health depends on the balance between osteoblast-driven formation and osteoclast-mediated resorption, which tightly regulates bone remodeling [1]. Among them, osteoblasts (OBs) are formed from mesenchymal stem cells through three stages, including the first stage of cell proliferation, which expresses collagen, transforming growth factor-beta (TGF-β), EGFR, and others. The second stage involves cell differentiation, which produces phosphatase, and the third stage involves cell mineralization, which promotes mineral deposition [2]. EGFR appears to be an important target protein for inhibiting and intervening in osteoporosis, as it is associated with cell migration, proliferation, and apoptosis [3,4]. As a typical transmembrane receptor, EGFR initiates a signaling cascade through ligand-induced dimerization, thereby activating TK and multiple downstream effector molecules. A variety of ligands activate EGFR, such as epidermal growth factor (EGF), heparin-binding EGF-like growth factor, transforming growth factor (TGF) -α, and so on [5]. EGF, one of seven ligands, detects extracellular signals by binding EGFR’s extracellular domain, triggering cell migration and proliferation [6]. Ligand binding induces EGFR dimerization, initiating signal transduction via conformational changes in the transmembrane (TM) domain, including TM helix dimerization or rearrangement [7]. In inactive EGFR, the TM domain dimerizes at the C-terminus, whereas ligand-free dimers form at the N-terminus. Transition between these states involves a 180° rotation of the TM helices. The two helices also undergo a 180° rotation between these states [8]. In addition, EGFR can undergo endocytic recycling and activate various intracellular signaling pathways, including RAS/RAF/MEK, PI3K/AKT/mTOR, and JAK/STAT [9,10]. Recent studies have found that EGFR overexpression can lead to osteoblast growth. As a result, significant efforts have been devoted to developing high-affinity EGFR-active compounds for the treatment and intervention of osteoporosis [11].

Multiple studies have demonstrated that active peptides exhibit strong affinity for EGFR, thereby regulating downstream cellular signaling pathways and participating in the stimulation of bone formation. These peptides can serve as effective active substances for the intervention of osteoporosis [3,4,12]. Lactoferrin peptides and bovine bone collagen peptides have been shown to bind to EGFR through hydrophobic interactions. Among them, lactoferrin peptides [13], duck protein-derived peptide VSEE [14], Japanese silkworm peptide SJP [15], and bovine gelatin hydrolysates [16] have been found to promote osteoblast differentiation by activating signaling pathways such as MAPK, Wnt-β, and others. In our previous studies, we found that soy yogurt exhibited strong osteogenic activity and could regulate periosteal proteins to activate the MAPK-signaling pathway to promote MC3T3-E1 cell differentiation [17,18]. The promotion of osteoblast differentiation by active peptides may be due to their ability to alter the conformation of EGFR upon binding, thereby activating intracellular signal transduction. However, how active peptides influence EGFR conformations remains unclear. Do active peptides block the binding of the natural ligand EGF to EGFR, or do they enhance the binding of EGF to EGFR and subsequently activate intracellular signaling? This question remains unresolved. Analyzing the relationship among active peptides, EGF, and EGFR, and elucidating the effects of active peptides on EGFR conformations, is crucial for understanding the mechanism by which active peptides regulate osteoblast differentiation.

To unravel the perplexing question of how active peptides influence EGFR conformation and thereby regulate bone metabolism, we isolate and purify a high-affinity EGFR peptide (HEP) from soy yogurt, firstly, while analyzing the osteogenic activity of HEP to promote osteogenic activity in vivo. Finally, the interaction between HEP and EGFR was detected by bioinformatics spectroscopy analysis and Western blot. Therefore, this study attempts to explore the Interactions between HEP peptide and EGFR involved in the osteoblast differentiation.

## 2. Materials and Methods

### 2.1. Reagents

Soybeans (CAAS, Beijing, China) were sourced from the China Academy of Agricultural Sciences and maintained under ambient conditions. The Lactobacillus plantarum strain was isolated and preserved in our laboratory facilities at China Agricultural University (Beijing, China). MC3T3-E1 cells were purchased from the National Infrastructure of Cell Line Resource (Beijing, China). α-MEM (alpha modification of Eagle’s minimum essential medium) and fetal bovine serum (FBS) were procured from Corning (Manassas, VA, USA). Other test kits were purchased from Solarbio Life Science (Beijing, China).

### 2.2. Antibodies

Primary antibodies targeting β-actin, EGFR, p-EGFR, PI3K, p-PI3K, and RUNX2, along with an HRP-conjugated goat anti-rabbit IgG (H+L) secondary antibody (1:2000), were acquired from Cell Signaling Technology (Manassas, VA, USA). All primary antibodies were diluted 1:1000 prior to use.

### 2.3. Preparation and Identification of Soybean Yogurt Peptides

#### 2.3.1. Preparation of Soy Yogurt

According to the description of Gan et al. [19], soybean yogurt was successfully prepared. Firstly, soybeans (150 g) were soaked overnight in 450 mL of distilled water at 25 °C and then crushed in a commercial stirrer with 12 times the volume of water for 5 min. The mixture was homogenized by a 220-bar high-pressure homogenizer, autoclaved at 115 °C for 15 min. Then, 10% (*v*/*v*) *Lactobacillus plantarum* was added and cultured at 37 °C for 12 h until the pH was decreased from 6.4 ± 0.04 to 4.64 ± 0.03. Secondly, SY-3 (<3 kDa) components were separated and collected. Finally, soybean yogurt components with the most significant osteogenic activity (SYO) and high EGFR affinity were isolated and identified by MTT and ALP assays.

#### 2.3.2. Identification of Peptides by UPLC-Q-TOF

The desalted SYO freeze-dried sample was dissolved in 0.1% formic acid–water solution, and the pH value was adjusted to 2–3. Separation employed a C18 column with mobile phases consisting of 0.1% (*v*/*v*) aqueous formic acid (A) and 0.1% (*v*/*v*) formic acid in acetonitrile (B). Injections of 10 μL were performed at a flow rate of 0.4 mL/min. According to Denovo scoring rules, a score greater than 50 is considered to be a valid identification result.

### 2.4. Screening of High EGFR Affinity Peptides

Peptides with Average Local Confidence (ALC) values ≥ 98 were selected for screening, and the sequences of these peptides were considered to be more reliable. A total of 25 peptide sequences (Table S1) were obtained. Molecular docking technology was used to evaluate the binding between peptides and EGFR molecules. The scoring function could be used to predict the strength of small molecule ligands binding to macromolecular receptors, which could be used to quickly screen out target substances in a large number of small molecules. The crystal structure of the EGFR protein (PDB ID: 1IVO) was sourced from the Protein Data Bank (https://www.rcsb.org/structure/1IVO accessed on 25 October 2024). Discovery Studio 4.5 (Dassault Systèmes BIOVIA, San Diego, CA, USA). was employed to generate three-dimensional structures of peptides while minimizing energy levels. Ligands and EGFR proteins were docked utilizing Discovery Studio, with the complex exhibiting the highest docking score deemed as the optimal docking result.

### 2.5. High EGFR Affinity Peptide Synthesis and Verification

The high EGFR affinity peptide (HEP) was purchased from Nanjing Peptide Biotechnology Co., Ltd. (Nanjing, China). The purified peptide was synthesized by solid-phase method. The purity of VVELLKAFEEKF was determined by high-performance liquid chromatography (HPLC) analysis (98%). 

### 2.6. Osteogenic Activity Analysis

#### 2.6.1. Cell Culture

MC3T3-E1 cells were purchased from the National Infrastructure of Cell Line Resource (Beijing, China). Cells were cultured in ɑ-MEM (containing 1% penicillin-streptomycin) with 10% FBS in an incubator containing 5% CO_2_ at 37 °C.

#### 2.6.2. Analysis of ALP Activity

MC3T3-E1 cells (3 × 10^5^/well) were plated in six-well plates and maintained in standard medium for 48 h, then induced to differentiate using medium containing 50 µg/mL ascorbic acid and 10 mM of β-glycerol phosphate for 4 days. Prior to HEP exposure at various concentrations (24 h, 37 °C), cells were pretreated with 20 µM of inhibitor for 2 h. After incubation, cells were rinsed with PBS and lysed using a PMSF-supplemented buffer and Bi Lon 92-II disruptor. ALP activity and total protein were assessed using colorimetric assays at 405 nm and 562 nm, respectively.

#### 2.6.3. Intracellular Ca^2+^ Concentration ([Ca^2+^]I) Measurement

Cells were cultured at a density of 2 × 10^5^ cells per well in a six-well culture plate at Duncan. After a 24 h treatment with HEP (1, 10, 100 μg/mL), the cells were harvested and resuspended in 1 mL of PBS. For intracellular calcium quantification, Flou-3/AM was introduced at a final concentration of 4 μmol/L. Subsequently, the cells were incubated for 30 min at 37 °C in a CO_2_ incubator shielded from light. Flow cytometry (Beckman Coulter, CA, USA) was utilized to determine the mean fluorescence intensity of cells in each group, with excitation and emission wavelengths set at 490 nm and 530 nm, respectively.

### 2.7. Analysis of HEP and EGFR Docking Mode

#### 2.7.1. Molecular Dynamics Simulation Analysis

All-atom MD simulations were performed using AMBER 22. GAFF2 and ff14SB force fields were applied to the ligand and protein. The system was solvated in a TIP3P box (10 Å), neutralized with Na^+^/Cl^−^, and minimized. After heating to 298.15 K in 200 ps, NVT (500 ps) and NPT (500 ps) equilibrations were followed by a 100 ns NPT production run. PME handled long-range electrostatics, SHAKE constrained hydrogen bonds, and Langevin dynamics (γ = 2 ps^−1^) controlled temperature. Pressure was set to 1 atm, timestep was set to 2 fs, and trajectories were saved every 10 ps.

#### 2.7.2. Receptor Domain Site-Directed Mutagenesis

The Calculate Mutation Energy (Binding) module in the Discovery Studio 4.5 (Dassault Systèmes BIOVIA, San Diego, CA, USA). was used for site-directed mutagenesis of the binding amino acid sites in the molecular docking results. The composite structure of EGFR and soybean peptide was pretreated and given.

#### 2.7.3. Binding Analysis of Peptides with EGFR Domain by Circular Dichroism (CD) Spectra

As previously mentioned by Deng et al. [20], peptides (0.3 mg/mL) were prepared in 100 mM of MOPS buffer (pH 7.0). Circular dichroism spectra (190–260 nm) were collected at 25 °C using a PiStar-180 spectrometer with a 1 mm pathlength cuvette, scanning at 50 nm/min with a 1 nm bandwidth and five accumulations. Mean residue ellipticity (θ) was calculated. Changes in peptide conformation upon exposure to Cellular or Mutant Cell Domains were analyzed, and secondary structure proportions were estimated using K2D2 softwarePiStar-180 spectrometer (Applied Photophysics, Leatherhead, UK).

### 2.8. Western Blot Assay

MC3T3-E1 cells were seeded at 2 × 10^5^; per well in six-well plates and allowed to adhere for 24 h. Cells were then incubated with HEP at concentrations of 1, 10, and 100 μg/mL for 24 h. After treatment, cells were harvested and lysed using 70 μL of cold lysis buffer supplemented with PMSF. Western blotting was performed following standard procedures. Protein signals were detected using an enhanced chemiluminescence system (Tanon, Shanghai) and quantified with ImageJ software (National Institutes of Health, Bethesda, MD, USA).

### 2.9. Statistical Analysis

All data were expressed as mean ± SD, and quantitative experiments were performed in triplicate. The statistical analysis was performed using the software package SPSS 17.0. One-way analysis of variance (ANOVA) with Tukey’s test was performed to compare groups. *p* values of less than 0.05 were considered statistically significant.

## 3. Results and Discussion

### 3.1. Isolation and Purification of Osteoblast-Activating Soybean Peptide from Soy Yogurt

Osteoporosis, the prevalent global bone ailment, is typically identified post-fracture. The disturbance of bone restructuring, encompassing bone resorption and formation, is a key factor in osteoporosis [21]. Recent endeavors have focused on enhancing bone formation and inhibiting resorption. Soy yogurt, created through liquid-phase fermentation with Lactic acid bacteria, is acknowledged as a promising agent for stimulating bone formation [22]. In this study, active substances that promote osteogenesis were first isolated and purified from soy yogurt. The results showed that the growth rate of cells was increased after treatment with 100 μg/mL of soy yogurt for 24 h, but there was no significant difference between the cells treated with 1 μg/mL or 10 μg/mL for 0 h in soy yogurt and the cells treated with soy yogurt for 24 h (Figure S1(A1–A3)). Compared with puree and 0 h of soy yogurt, 12 h of soy yogurt showed higher osteogenic activity in a time-dependent manner. Meantime, the osteogenic activities of the cells treated with 100 μg/mL of puree, 0 h of soy yogurt, and 12 h of soy yogurt were significantly increased compared with those of the control at 24, 48, and 72 h. Furthermore, an increase of 9.534%, 16.280%, and 18.691% in cell proliferation was observed after 24, 48, and 72 h of 100 μg/mL treatment. However, there was no significant difference between the 1 μg/mL and 10 μg/mL dose groups.

Cell differentiation is the crucial step in bone formation. During the differentiation process, the activity of osteoblast differentiation marker ALP directly reflects the activity and/or function of osteoblasts [23]. Therefore, to investigate the effect of soy yogurt on cell differentiation, the changes of ALP activity caused by soybean milk, 0 h of soy yogurt, and 12 h of soy yogurt were analyzed. The results showed that the activity of ALP in MC3T3-E1 cells increased from 0.0058 U/mg to 0.0087 U/mg after treatment with 100 μg /mL for 12 h in yogurt. The concentration of ALP in cells treated with 12 h of yogurt was higher than that in cells treated with soybean milk and 0 h of yogurt. This indicated that fermentation increased the differentiation of MC3T3-E1 (Figure S1C).

To further seek the peptides with high osteogenesis, the 12 h of soy yogurt was separated into three fractions (>10 KD, 3–10 KD, and <3 KD), which were then evaluated by the MTT and ALP assay. As shown in Figure S1(B1–B3), the cell proliferation increased by 5.937%, 14.808%, and 21.288%, respectively, after 72 h of treatment with 100 μg/mL of the three parts. In addition, the activity of <3 KD component was significantly higher than that of other groups. After treatment with 1, 10, and 100 μg/mL of this component for 72 h, the cell proliferation increased by 3.973%, 17.736%, and 21.288%, respectively. This was consistent with previous reports, indicating that smaller peptides had higher osteogenesis.

Furthermore, the ALP activity of <3 KD fraction was investigated. As shown in Figure S1C, the activity of ALP in MC3T3-E1 cells was increased from 0.0058 U/mg to 0.0144 U/mg after treatment with 100 μg/mL < 3 KD fractions. These results indicated that the <3 KD fraction possessed a potential pro-osteogenic function and required further separation and purification of active peptides.

### 3.2. Isolation of Peptides and Identification by UPLC-Q-TOF

To further isolate high EGFR affinity peptides, the Dextran gel (Sephadex G–15) chromatography was used to separate the soybean peptides from <3 KD fractions. As shown in Figure S2, four fractions (P1–P4) were reaped. Compared with other components P1, P2, and P3, P4 showed the highest MTT and ALP activity.

**Figure 1:**
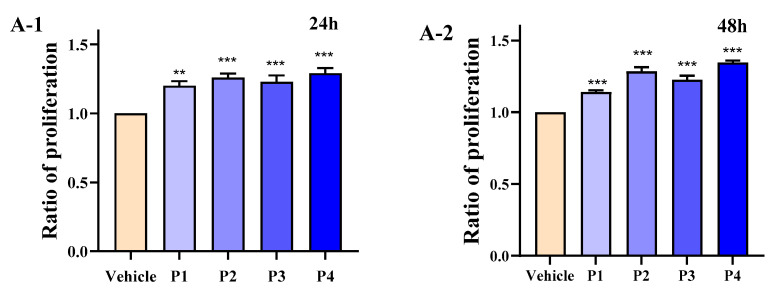

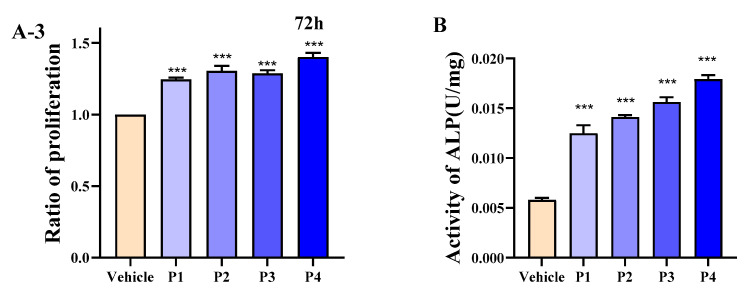
Effect of four fractions (P1–P4) on the proliferation of osteoblasts after purification by Sephadex G–15 column. (**A**) The effect of four fractions on osteoblast proliferation; (**B**) The effect of four fractions on ALP activity. Means ± SEMs represent the data and were analyzed by the one-way analysis of variance (ANOVA) method, followed by Tukey’s multiple comparison test, where n = 3. Statistical significance was determined at ** *p* < 0.01, and *** *p* < 0.001, compared with the vehicle.

The cell proliferation rate was increased by 29.132%, 34.667%, and 40.178% after 24, 48, and 72 h of P4 treatment. Moreover, the activity of ALP in P4 treated cells was 0.0179 U/mL (Figure 1A1–A3,B). Finally, the P4 was then analyzed using UPLC-Q-TOF, and the amino acid sequences of the polypeptides were tested. It was found that the molecular weight of P4 peptides was between 311.1894 Da and 540.3026 Da, and the MS/MS identification scores of 25 peptides were greater than 98%. Therefore, the properties of the peptide with the best activity among these 25 peptides needed to be analyzed, including their sequence, pI value, net charge, Hydrophobic uncharged, Grand average of hydropathicity, and Aliphatic amino acid index. This inference was supported by previous studies on the hydrolysis of Mytilus edulis, where most peptides with high osteogenic activity had MWs lower than 1500 Da [24].

### 3.3. Screening of Peptides with High EGFR Binding Ability

To isolate and identify EGFR high-affinity peptides, molecular docking simulations were performed using Discovery Studio 2019 (BIOVIA, San Diego) with the CDOCKER module, where binding free energies (ΔG, kcal/mol) were calculated to quantitatively evaluate peptide–EGFR interactions. In Table 1, the calculated -CDOCKER energy values were listed. The higher scores result in the higher binding affinity [25,26]. Based on complementary docking scores and MM/GBSA binding energy calculations, we identified five high-affinity candidate peptides for synthesis and experimental validation. Subsequent ALP activity assays confirmed their bioactivity, with peptide HEP in P4 demonstrating superior EGFR binding affinity among all synthesized peptides.

### 3.4. HEP Stimulates Differentiation and Intracellular Calcium of MC3T3-E1 Cells

**Figure 2:**
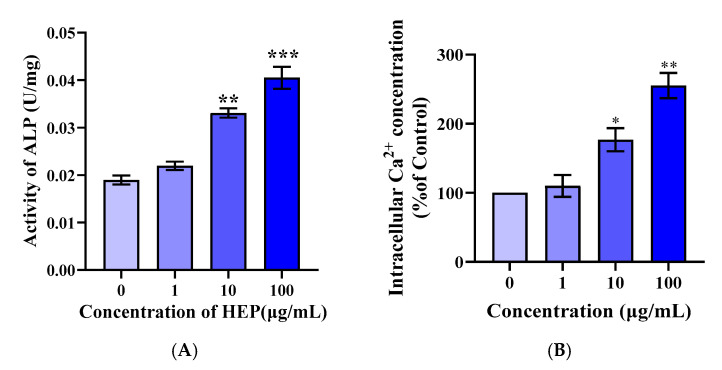
Effect of HEP on promoting osteogenesis. (**A**) Effect of HEP (1, 10, and 100 μg/mL) on the activity of ALP; (**B**) Effects of soybean peptides on the concentration of intracellular Ca^2+^. Means ± SEMs represent the data and were analyzed by the one-way analysis of variance (ANOVA) method, followed by Tukey’s multiple comparison test, where *n* = 3. Statistical significance was determined at * *p* < 0.05, ** *p* < 0.01, and *** *p* < 0.001, compared with the vehicle.

To confirm the osteogenic potential of HEP, a synthetic peptide matching its sequence (98% purity) was prepared. ALP, a key osteoblast marker linked to phosphate supply and mineralization, was used to assess activity [26,27]. Following HEP treatment (1–100 μg/mL), ALP activity in MC3T3-E1 cells significantly increased from 0.01897 ± 0.00165 to 0.04051 ± 0.00402 U/mg in a concentration-dependent manner (*p* < 0.001), indicating enhanced osteogenic differentiation (Figure 2A).

The calcium-transforming function of osteoblasts is essential for bone metabolism. Intracellular calcium, as a second messenger, participates in the regulation of cellular proliferation and protein activation. Some studies have shown that cytoplasmic calcium is associated with pre-osteoblast proliferation and differentiation [28]. To determine the effect of HEP on pre-differentiation and mineralization of osteoblasts by promoting calcium influx, intracellular calcium concentration was monitored. Meantime, HEP elevated the concentration of intracellular calcium in a dose-dependent manner (shown in Figure 2B), indicating that HEP was involved in the regulation of cellular signaling pathways, thereby promoting osteoblast differentiation. It was suggested that HEP could bind with receptors on the osteoblast membrane and induce an influx of Ca^2+^ and the activation of cells.

### 3.5. Interactions Between the Peptides and EGFR

#### 3.5.1. Molecular Docking Binding Site Analysis

**Figure 3:**
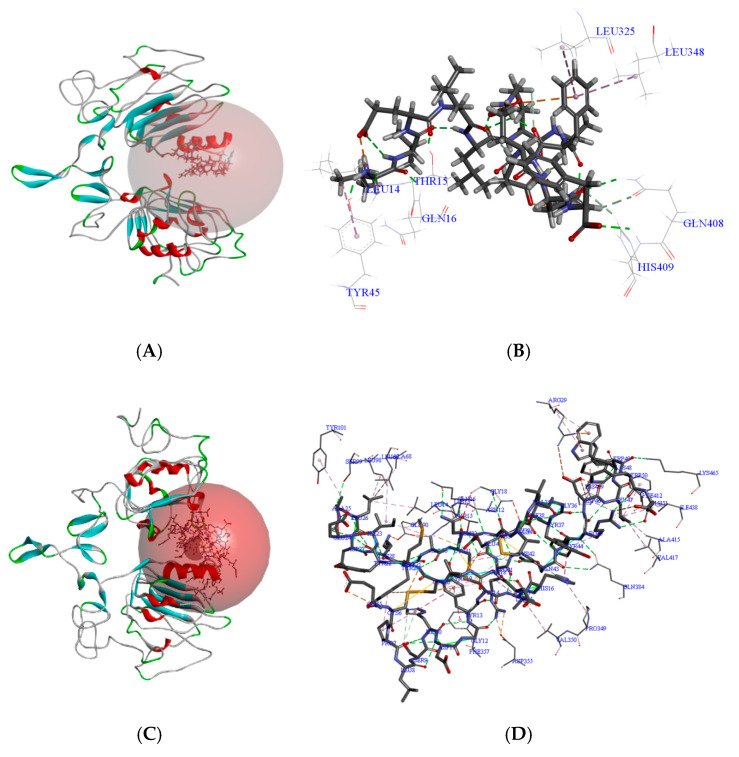
Docking calculations for the interaction of HEP and EGF with EGFR. (**A**) HEP and EGFR molecular docking position and 3D binding structure; (**B**) HEP and EGFR molecular docking amino acid interaction sites; (**C**) EGF and EGFR molecular docking position and 3D binding structure; (**D**) EGF and EGFR molecular docking amino acid interaction sites.

The binding of HEP to EGFR was examined (Figure 3A). Key hydrogen-bonded amino acids involved in this interaction include GLU3, LYS4, GLN8, SER11, ASN12, ASN40, ALA265, ARG285, HIS346, GLN408, and HIS409 (Figure 3C). Non-covalent interactions such as hydrogen bonds, hydrophobic, and electrostatic forces were observed. Notable amino acids for hydrogen bonding with EGFR were LYS13, THR15, GLN16, GLY18, and ASP22. The attractively charged amino acids of HEP for the interaction with EGFR included ARG29 and LYS13. In addition, the Π- Cation, alkyl interaction, and Π- Alkyl interaction amino acids of HEP and EGFR had ASN12, LEU14, and LEU25. Therefore, the interaction of peptide HEP with EGFR may play a role in promoting the activity of osteoblast proliferation, which has never been reported previously, to the best of our knowledge.

Notably, several HEP–EGFR interaction sites overlap with those of EGF, including ASN12, LYS13, GLN16, GLY18, ASP22, HIS346, and GLN408. Additionally, HEP binds uniquely to ARG285, GLN8, and HIS409, matching sites reported for South Asian wild mud and fermented milk peptides by Wu et al. [29]. Therefore, HEP might activate EGFR and promote osteoblast proliferation like EGF. The interaction between HEP and EGFR is different from the previously reported long peptide with 31 amino acid residues [30]. The regulation of EGFR by HEP is similar to that of TGF-α, playing a complementary role rather than a competitive binding.

Both physicochemical and structural properties affect the activity of osteogenic peptides. As the membrane-bound proteins, the position of their amino acid sequence in the lipid bilayer depended on their hydrophobic areas. In general, the GRAVY positive (hydrophilic) values and GRAVY negative (hydrophobic) values were used for the characterization of peptides. In contrast, a GRAVY score below 0 would be related to a hydrophilic rounded structure, and a score above 0 would be very likely to become a hydrophobic membrane-bound system. According to Table 1, Nos. 1, 3, 4, 7, 11, 12, 13, 14, 15, 16, 17, 19, 20, 21, 22, 23, 24, and 25 peptides showed positive GRAVY values, suggesting the hydrophilic characters, while others showed negative values, indicating more hydrophobic surfaces. Moreover, the stability and bioavailability of peptides are usually highly related to peptide properties, such as hydrophilicity, charge, and size. Typically, peptides that are highly hydrophilic and negatively charged are more easily transported through small intestine cells through tight junctions to improve the bioavailability of peptide. The HEP peptide is highly hydrophilic and contains three negatively charged residues, potentially contributing to its bioavailability. This is consistent with Yang M’s reports.

#### 3.5.2. Molecular Dynamics Simulations

**Figure 4:**
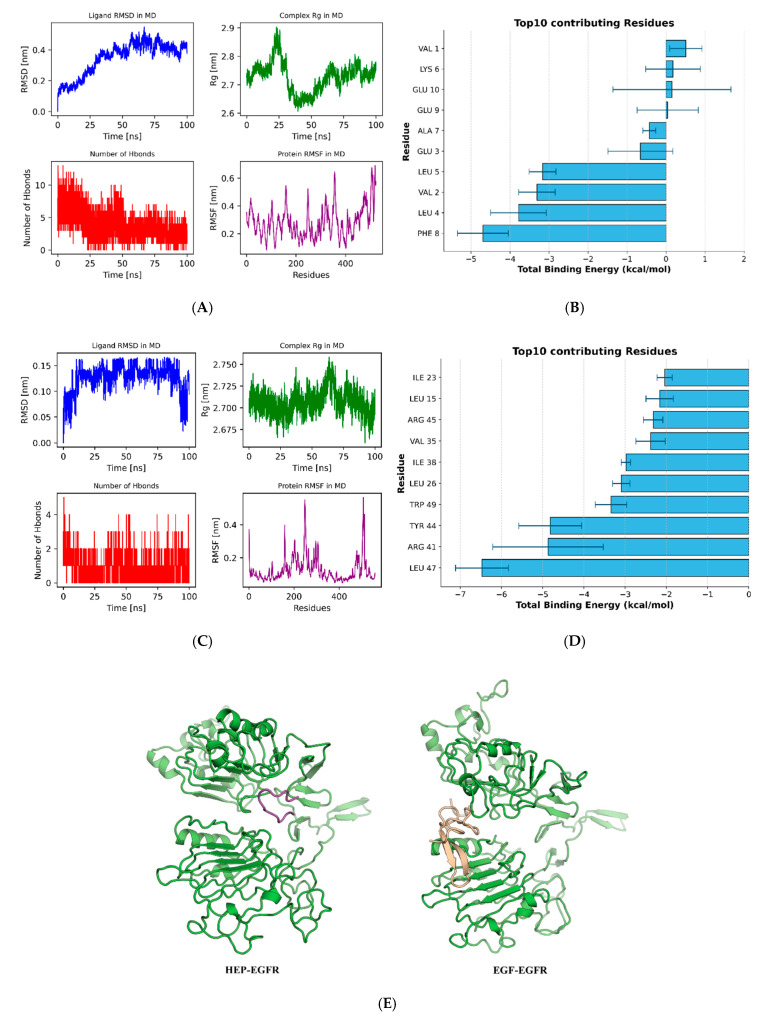
The interaction results of HEP, EGF, and EGFR during 100 ns molecular dynamics simulations. (**A**) Root mean square deviation (RMSD), radius of gyration (Rg), number of hydrogen bonds (Hbonds), Root mean square fluctuation (RMSF) of HEP and EGFR; (**B**) The binding energy contributed to the top 10 amino acids of HEP and EGFR. (**C**) RMSD, Rg, number of Hbonds, RMSF of EGF and EGFR; (**D**) The binding energy contributed to the top 10 amino acids of EGF and EGFR; (**E**) Conformations of ligands and EGFR at 100 ns.

Due to the limitation of rigid binding of molecular docking, it may lead to false positive results [31]. Molecular dynamics (MD) was used to verify the binding ability of HEP and EGFR. Therefore, 100 ns of MD simulation was used to study the binding stability, and a detailed interaction curve was obtained, as shown in Figure 4A.

By analyzing the changes of RMSD in HEP and EGFR complexes, it could be observed that during the simulation process of 100 ns, the RMSD of HEP-EGFR showed a continuous upward trend in the initial stage, reached a plateau at about 50 ns, and finally stabilized at about 0.4–0.5 nm. This indicated that the polypeptide gradually completes the conformational adaptation in the binding site and maintains a relatively stable binding state in the later stage, with a certain conformational stability. In addition, the curve of the complex of EGF and EGFR gradually balanced the system from 10 ns, with an average RMSD value of 0.10–0.15 nm, respectively. These findings suggested all complexes bind the active site and achieve stability at different rates, with higher activity peptides displaying faster convergence and lower RMSD values.

The number of hydrogen bonds between HEP and EGFR was more in the early stage of the simulation, up to 12, then the overall trend was downward, and the number of hydrogen bonds between HEP and EGFR fluctuated between 2–6 in the later stage. This might reflect the rapid and stable binding of peptides through more hydrogen bonds in the early stage, while the number of hydrogen bonds decreased but still maintained a certain level in the later stage, suggesting that the binding mode tended to be optimized and stable.

The RMSF analysis of protein residues showed that the atomic fluctuation range of most regions was between 0.2–0.4 nm, and only the terminal region or some flexible ring regions fluctuated greatly, with a maximum of more than 0.6 nm. This indicated that the overall skeleton of the protein is stable, and only a few regions have certain flexible changes, which might be involved in peptide binding or conformational regulation. Therefore, molecular dynamics simulations demonstrated the stability of the binding between HEP and EGFR.

#### 3.5.3. Amino Acid Site-Directed Mutagenesis

**Figure 5:**
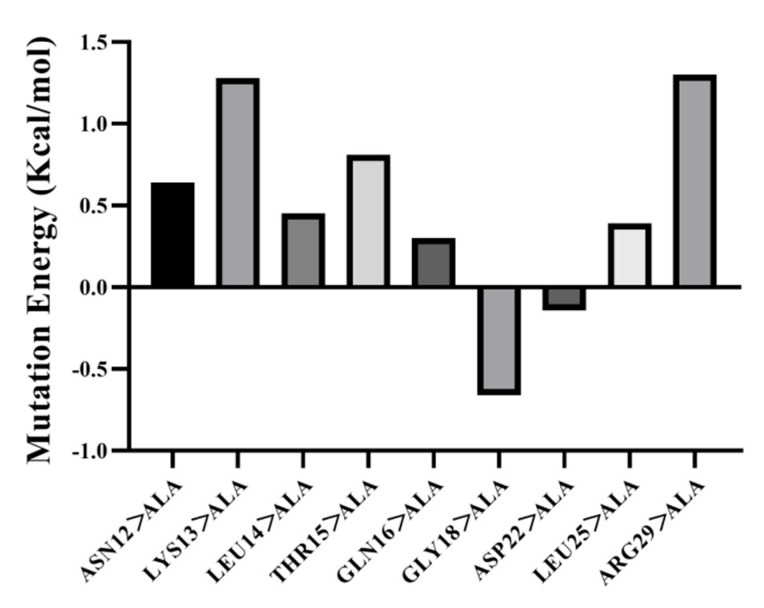
The binding energy change of HEP after the amino acid of the molecular docking binding site on EGFR was mutated to Ala.

To determine the relationship between the structure of EGFR and the interaction of HEP ligands, site-directed mutagenesis of key amino acids for ligand binding was performed (shown in Figure 5). LYS13, THR15, GLN16, GLY18, ASP22, ARG29, ASN12, LEU14, and LEU25 in the EGFR structure were mutated to alanine. The mutation results were evaluated by mutation energy. Mutation energies between −0.5 and +0.5 indicate minimal impact on affinity. Criteria for evaluation specify mutation energy within this range, signifying negligible effect on affinity. Mutation energies exceeding 0.5 result in reduced affinity and interaction between receptor and ligand. Conversely, energies below −0.5 enhance affinity and interaction. The results showed that the mutation energies of ASN12, LYS13, THR15, and ARG29 were all greater than 0.5, resulting in a decrease in affinity. The mutation energies of LEU14, GLN16, ASP22, and LEU25 were between −0.5 and 0.5, and these amino acids had no significant effect on affinity. Only the mutation energy of GLY18 was less than −0.5, as shown in Figure 4. Therefore, it was speculated that ASN12, LYS13, THR15, and ARG29 were the key amino acids of HEP binding to the EGFR receptor. Meanwhile, the HEP and EGF functional interplay may manifest as either competitive inhibition or allosteric synergy, with critical implications for osteogenic signaling. Bioactive peptides act as allosteric modulators, altering the conformation of EGFR or stabilizing the EGF-EGFR complex, enhancing downstream signaling, like Pilose antler peptide. However, bone collagen peptides that reduced EGFR phosphorylation could attenuate downstream MAPK/ERK signaling, potentially suppressing osteoblast proliferation. Therefore, the synergistic engagement of HEP and EGF with EGFR potentiates osteoblast proliferation, differentiation, and bone metabolism through amplified downstream signaling, ultimately enhancing bone mineral density (BMD) and mitigating osteoporotic deterioration in vivo.

### 3.6. Analysis of the Peptide-Binding Activity of the EGFR

Based on molecular docking results, HEP could bind to the extracellular domain of EGFR, which is similar to that observed between EGF and EGFR. To verify our speculation regarding the binding capacity of HEP with EGFR, the circular dichroism was used to analyze the interaction between HEP, the extracellular domain protein (EDP), and Mutant extracellular domain proteins (MEDP), where the binding sites in the extracellular domain were mutated and cannot bind to HEP.

**Figure 6:**
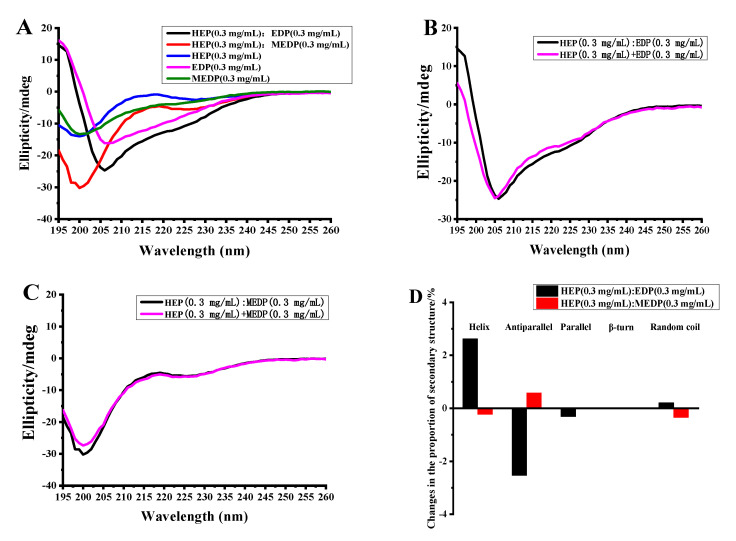
CD spectra of HEP with extracellular domain protein (EDP) and Mutant extracellular domain proteins (MEDP). (**A**) CD spectra of HEP, EDP, MEDP, HEP in the presence of EDP, and HEP in the presence of MEDP; (**B**) CD spectra and data addition comparison of HEP and EDP mixed samples; (**C**) CD spectra and data addition comparison of HEP and MEDP mixed samples; (**D**) The second structure ratio of samples and their mixture.

The results showed that the secondary structures of HEP and MEDP are mainly β Folding and irregular curling, while the secondary structure of EDP is mainly composed of α Spiral and irregular curls (shown in Figure 6A).

The secondary structures could change after mixing, when two substances can interact. Consequently, significant shifts in signal peaks will be observed in the circular dichroism (CD) spectra within the same wavelength range. To be sure whether HEP truly interacts with the structural domain of EGFR, the secondary structures of HEP, EDP, and an equimolar mixture of HEP and EDP were analyzed separately. The results showed that there have been significant shifts in the CD data between the sum of their individual structures and the equimolar mixture of HEP and EDP, indicating an interaction between HEP and EDP. However, the mixed solution of HEP and MEDP showed no significant change compared to the spectral lines of the addition data, and the signal peak wavelength did not shift, indicating that there was no interaction between HEP and MEDP. The peak shape changes around 200 nm were caused by a significant increase in the total concentration of the mixed solution. The results are shown in Figure 6C.

Figure 6D showed the difference between the proportion of various secondary structures in the mixed solution and the proportion of the addition data graph obtained by analyzing the mixed solution and the addition data graph using CDNN. It could be seen that the changes in the mixed solution of samples HEP and EDP were significantly stronger than those in the mixed solution of samples HEP and MEDP (shown in Figure 6B). The proportion change of mixed solutions of HEP and MEDP was less than 1%, indicating almost no interaction between HEP and MEDP. The proportion of helical and antiparallel folding structures in the mixed solutions of 1 and 2 showed a change of over 2%, indicating an interaction between HEP and MEDP, which was consistent with the conclusion of spectral analysis.

### 3.7. HEP Induced the Activation of EGFR/PI3K/RUNX2 Pathways in MC3T3-E1 Cells

**Figure 7:**
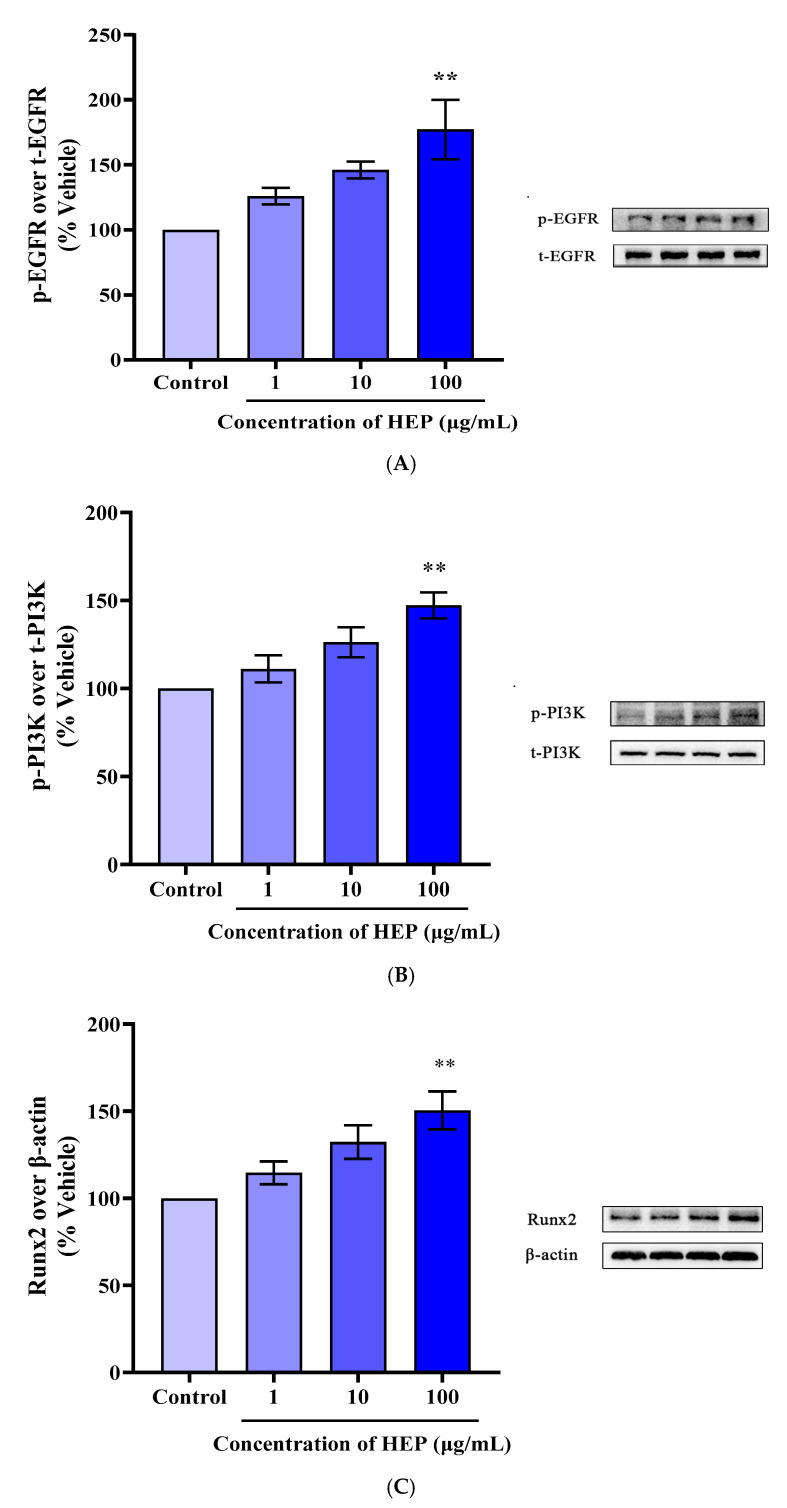
Effect of HEP on EGFR/PI3K/Runx2 Pathway activation in MC3T3-E1 cells. (**A**) Effect of different concentrations of HEP on EGFR protein expression in MC3T3-E1 cells; (**B**) Effect of different concentrations of HEP on PI3K protein expression in MC3T3-E1 cells; (**C**) Effect of varied concentrations of HEP on Runx2 protein expression in MC3T3-E1 cells. Means ± SEMs represent the data and were analyzed by the one-way analysis of variance (ANOVA) method, followed by Tukey’s multiple comparison test, where *n* = 3. Statistical significance was determined at ** *p* < 0.01, compared with the vehicle.

Cell proliferation can be regulated by epidermal growth factor and epidermal growth factor receptor (EGFR) [9]. To verify whether the effect of HEP on osteogenic activity is exerted by interacting with EGFR, we analyzed the total protein and phosphorylation levels of EGFR after adding different concentrations of HEP. Western Blotting was used to detect the expression of EGFR in MC3T3-E1 cells. Figure 7A showed that compared with the vector group, 100 μg/mL of HEP treatment significantly increased the phosphorylation of EGFR in MC3T3-E1 cells (*p* < 0.01). These results indicated that HEP could interact with EGFR and regulate its downstream signaling pathway to promote osteogenic activity [32]. The PI3K pathway is one of the main downstream signaling pathways of EGFR [33]. A large number of studies have shown that activation of PI3K-related signaling pathways could promote osteoblast proliferation and differentiation, thereby inhibiting osteoporosis [34]. Figure 7B demonstrated that 100 μg/mL of HEP treatment significantly increased the phosphorylation of PI3K in MC3T3-E1 cells (*p* < 0.01). Runx2 (Runx-related transcription factor 2) is one of the specific transcription factors of osteogenic differentiation, which is a key factor in the differentiation of mesenchymal stem cells into osteoblasts [35,36]. Runx2 overexpression promoted cell differentiation, while its downregulation caused osteoblast maturation arrest. Runx2 significantly regulates osteoblast differentiation. Figure 7C showed that Runx2 phosphorylation was significantly increased by 50.43% (*p* < 0.01) after MC3T3-E1 cells were treated with 100 μg/mL of HEP for 24 h.

## 4. Conclusions

HEP, a high EGFR affinity soybean peptide, showed strong osteogenic activity in vitro. In this study, bioinformatics, spectroscopy analysis, and Western blot were applied to analyze the interaction between HEP and EGFR. The above findings indicated that HEP exhibits different loop conformation and forms complementary binding sites with EGF during the binding with EGFR. These results indicated that HEP might play a role in promoting bone remodeling, thereby enhancing the interaction between EGF and EGFR. Furthermore, it could be considered a potential active factor for the development of functional foods aimed at preventing osteoporosis. However, these results are in vitro. We could delineate the in vivo anti-osteoporotic potential of bioactive peptides in the future and elucidate their crosstalk with pivotal bone remodeling pathways, particularly RANKL-mediated osteoclastogenesis and Wnt-driven osteogenesis.

## Figures and Tables

**Table 1:** Sequence, Localization, and physicochemical properties of P4 peptide.

								Amino Acid Distribution (%)								
No.	*m/z*	Scan	z	AA Sequence	*pI*	Net Charge	-CDOCKER ENERGY	Hydrophobic Uncharged	Acidic	Basic	Others	Aromatic	Aliphatic Amino Acid Index	GRAVY	Permeability	Peptide Ranker Score
1	484.61	16,182	3	VVELLKAFEEKF	4.48	−1	184.077	58.3	25	16.7		16.7	121.67	0.425	0.210606	0.253008
2	504.7534	7322	2	ATAGDEGKLF	4.18	−1	143.983	40	20	10	30	10	59	−0.22	0.147783	0.407089
3	540.3026	11,560	2	LFEESLKTL	4.26	−1	138.631	44.4	22.2	11.11	22.2	11.11	130	0.2	0.185338	0.206268
4	338.8695	4925	3	AVGGLGKLGK	9.54	1	149.434	36.36	9.09	18.18	36.36	-	106.36	0.064	0.386555	0.341914
5	532.2457	13,585	2	NGDDLFVHF	4.11	-2	114.448	44.44	22.22	11.11	22.22	22.22	75.56	−0.056	0.059725	0.800047
6	473.8885	4251	3	EDDEQLPSHPP	4.10	-3	130.008	8.33	33.33	16.67	41.67	-	32.5	−2.25	0.0890831	0.37201
7	518.7401	13,601	2	SGDDLFVFH	4.11	-2	131.248	44.44	22.22	11.11	22.22	22.22	75.56	0.244	0.0539904	0.815582
8	521.7354	10,322	2	WFNDEKGF	4.18	−1	140.384	37.5	25	12.5	25	37.5	0	−1.263	0.0730926	0.8241
9	391.5593	3681	3	VSETGKLVPSR	9.70	1	131.957	27.27	9.09	18.18	45.45	-	88.18	−0.364	0.289799	0.122207
10	315.19	4360	3	VGGLGKLGKD	9.54	1	135.765	30	10	20	40	-	107	−0.11	0.361272	0.279506
11	425.2395	7354	2	LLKAFEE	4.26	−1	114.8	57.14	28.57	14.29	-	14.29	125.71	0.186	0.210617	0.126237
12	365.2287	5542	2	LLNLEK	6.41	0	98.5768	50	16.67	16.67	16.67	-	195	0.083	0.550142	0.0947654
13	426.2471	6197	2	LVELYSK	6.40	0	108.511	57.14	14.29	14.29	14.29	14.29	152.86	0.329	0.220626	0.073327
14	365.2286	6662	2	VELKLQ	6.41	0	102.062	16.67	16.67	16.67	16.67	-	178.33	0.15	0.535372	0.0609186
15	350.7338	6859	2	VVELLK	6.41	0	100.583	33.33	16.67	16.67	-	-	226.67	1.433	0.610505	0.0667024
16	336.7177	6312	2	LLADLK	6.34	0	95.7294	66.67	16.67	16.67	-	-	211.67	0.967	0.548048	0.228773
17	460.2653	6597	2	LAGALPSYK	9.30	1	93.3458	55.56	-	11.11	33.33	11.11	108.89	0.356	0.236381	0.435549
18	340.2099	2603	2	GFKSLK	10.81	2	87.2354	33.33	-	33.33	33.33	16.67	65	−0.4	0.262743	0.48218
19	332.1945	8994	2	FDLLR	6.34	0	78.0346	60	20	20	-	20	156	0.48	0.366771	0.750974
20	343.7315	6976	2	SVVLLR	10.55	1	78.4668	66.67	-	16.67	16.67	-	226.67	1.783	0.626641	0.247147
21	381.2316	6090	2	LLPYGKA	9.30	1	79.483	57.14	-	14.29	28.57	14.29	125.71	0.314	0.332674	0.349653
22	339.7	5373	2	AFSRVV	10.55	1	75.3562	66.67	-	16.67	16.67	16.67	113.33	1.283	0.267797	0.293781
23	311.1894	5862	2	AVRLY	9.35	1	67.5503	60	-	20	-	20	156	0.8	0.578736	0.293136
24	335.2177	4103	2	LPALQK	9.70	1	68.0429	50	-	16.67	33.33	-	146.67	0.067	0.589914	0.235953
25	331.6788	7513	2	TLFGPQ	6.10	0	63.0582	33.33	-	-	66.67	16.67	65	0.067	0.126841	0.42418

## Data Availability

All related data and methods are presented in this paper and the Supplementary Materials. Additional inquiries should be addressed to the corresponding author.

## Supplementary Materials

The following supporting information can be downloaded at: https://www.mdpi.com/article/10.3390/foods14173032/s1, Figure S1: Effects of fermented yogurt and its isolation on MTT and ALP activity in MC3T3-E1. A-1, A-2, A-3: Pure, Soy yogurt 0 h, Soy yogurt 24 h at concentrations of 0.1, 10, 100 μg/mL was tested for MC3T3-E1 cells at 24, 48 and 72 h. B-1, B-2, B-3: Different fractions samples (>10 KD, 3–10 KD, <3 KD) at concentrations of 0.1, 10, 100 μg/mL was tested for MC3T3-E1 cells at 24, 48 and 72 h. C: Effect of different samples (pure, soy yogurt 0 h, soy yogurt 24 h, >10 KD, 3–10 KD, <3 KD) on alkaline phosphatase activity(ALP) at 24 h with 100 μg/mL. * *p* < 0.05, ** *p* < 0.01, and *** *p* < 0.001 vs. Vehicle; Figure S2: Elution diagram of the Sephadex G-15 column. Elution was monitored at wavelengths of 215, 220, and 280 nm. The peaks are numbered P1–P4; Figure S3: A: Identification of HEP (VVELLKAFEEKF) by UPLC-Q-TOF; B: MS/MS mass spectrum of dodecapeptide HEP; Table S1: Volume and accessible surface area to water (ASA) of peptides; Table S2: Binding free energies and energy components predicted by MM/GBSA (kcal/mol).

## Abbreviations

HEP	soy peptide
VVELLKAFEEKF	Val-Val-Glu-Leu-Leu-Lys-Ala-Phe-Glu-Glu-Lys-Phe
EGFR	epidermal growth factor receptor
EGF	epidermal growth factor
ALP	cellular alkaline-phosphatase
CD	circular dichroism
RMSD	Root mean square deviation
Rg	radius of gyration
Hbond	number of hydrogen bond
RMSF	Root mean square fluctuation
