# Supplementary material for: Interactions Between HEP Peptide and EGFR Involved in the Osteoblast Differentiation
Source: Foods. 2025 Aug 29;14(17):3032. doi: 10.3390/foods14173032 (PMC12427943; doi:10.3390/foods14173032)
Supplement: Supplementary file 1 [file foods-14-03032-s001.zip › foods-3816668-supplementary.pdf]

Supplementary Materials

Table S1. Volume and accessible surface area to water (ASA) of peptides.

Peptide(no.)	AA Sequence	ASA	Type of surface (% of total ASA)			
			Hydrophobic	Polar	Negative	Positive
			(ASA_H)	(ASA_P)	(ASA^-)	(ASA^+)
1	VVELLKAFEEKF	1723.7797	1005.8078	717.9718	596.5970	1127.1826
2	ATAGDEGKLF	1315.9900	648.2913	667.6987	536.0781	779.9119
4	AVGGLGKLGK	1266.9324	562.8857	704.0467	418.1541	848.7783
8	WFNDEKGF	1340.5574	748.1027	592.4546	555.1332	781.0144

Table S2. Binding free energies and energy components predicted by MM/GBSA (kcal/mol).

Ligands	ΔE_{VEW}	ΔE_{elec}	ΔG_{GB}	ΔG_{SA}	ΔG_{bind}
AFSRVV	-23.19	-73.83	84.13	-3.66	-16.55
ATAGDEGKLF	-33.33	-42.89	60.16	-4.09	-20.15
AVGGLGKLGKD	-39.84	-58.22	72.26	-5.45	-31.26
AVRLY	-23.53	-13.28	29.70	-3.21	-10.32
EDDEQLPSHPPR	-57.07	-160.32	203.37	-7.76	-21.78
FDLLR	-25.06	-86.15	99.92	-3.90	-15.19
GFKSLK	-22.84	-76.87	82.18	-3.93	-21.47
LAGALPSYK	-29.54	-39.58	54.43	-4.29	-18.98
LFEESLKTL	-35.03	-61.71	89.47	-4.98	-12.24
LLADLK	-31.57	-36.43	54.59	-3.53	-16.94
LLKAFEE	-22.66	-54.09	64.62	-3.69	-15.83
LLNLEK	-22.50	-50.49	61.44	-3.59	-15.13
LLPYGKA	-26.29	-53.13	66.79	-3.88	-16.51
LPALQK	-28.64	-19.92	25.77	-3.76	-26.54
LVELYSK	-22.52	-45.99	65.79	-5.06	-5.78
NGDDLFVHF	-31.33	-67.62	95.06	-4.67	-8.56
SGDDLFVFH	-28.77	-82.21	93.53	-3.84	-21.28
SVVLLR	-22.07	-18.67	28.77	-3.12	-15.09
TLFGPQ	-27.27	-13.77	37.38	-3.13	-6.78
VELKLQ	-29.64	-22.44	37.71	-3.80	-18.17
VGGLGKLGK	-32.07	-25.70	48.06	-4.19	-13.90
VSETGKLVPSR	-45.87	-2.50	48.27	-5.96	-6.07
VVELLK	-24.20	-47.53	55.46	-3.78	-20.05
VVELLKAFEEKF	-44.82	-134.06	161.05	-6.83	-24.67
WFNDEKGF	-47.71	-67.79	102.93	-6.18	-18.75

ΔE_{vdW}: van der Waals energy.

ΔE_{elec}: electrostatic energy.

ΔG_{GB}: electrostatic contribution to solvation.

ΔG_{SA}: non-polar contribution to solvation.

ΔG_{bind}: binding free energy.

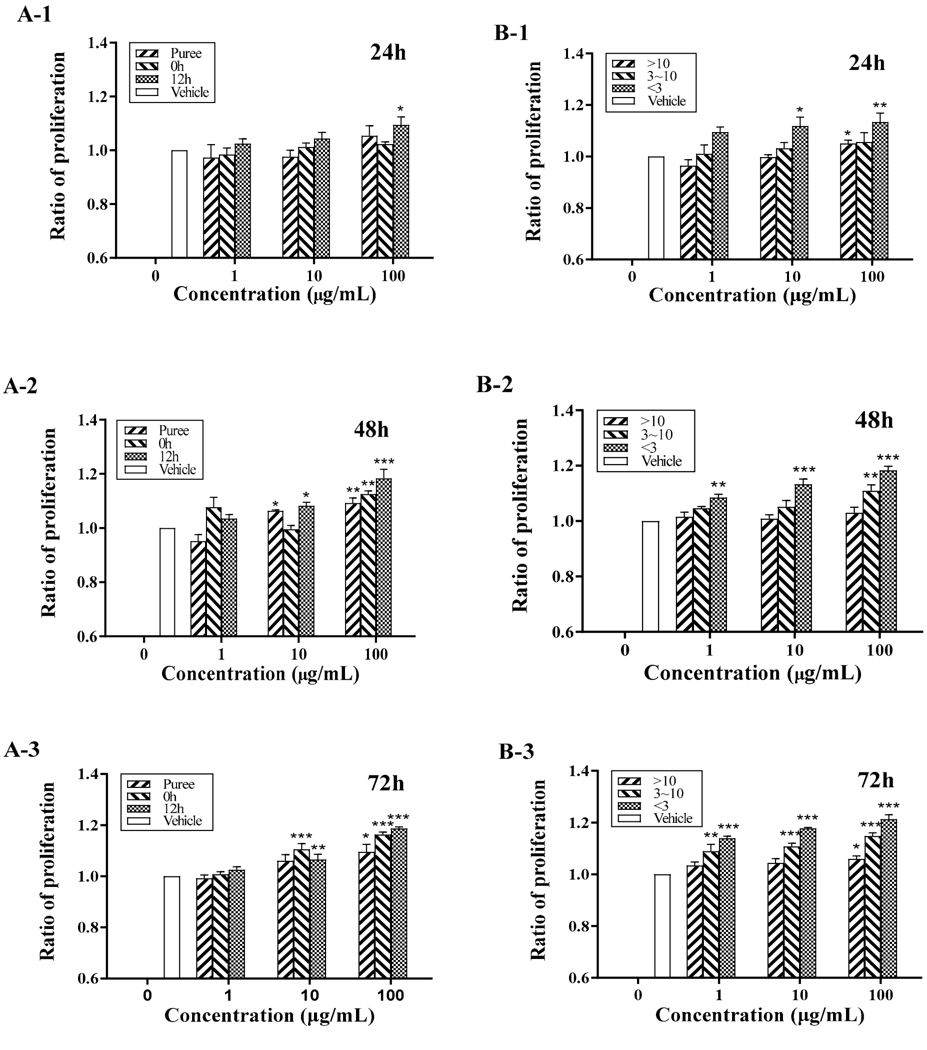

C

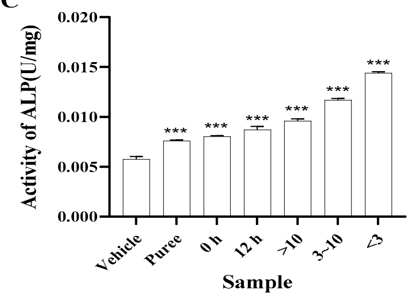

Figure S1 Effects of fermented yogurt and its isolation on MTT and ALP activity in MC3T3-E1. A-1, A-2, A-3: Pure, Soy yogurt 0h, Soy yogurt 24h at concentrations of 0.1,10,100 μg/mL was tested for MC3T3-E1 cells at 24, 48 and 72 h. B-1, B-2, B-3: Different fractions samples (>10KD,3-10KD, <3KD) at concentrations of 0.1,10,100 μg/mL was tested for MC3T3-E1 cells at 24, 48 and 72 h. C: Effect of different samples (pure, soy yogurt 0h, soy yogurt 24h, >10KD,3-10KD, <3KD) on alkaline phosphatase activity(ALP) at 24h with 100μg/mL. * $p < 0.05$, ** $p < 0.01$, and *** $p < 0.001$ vs. Vehicle.

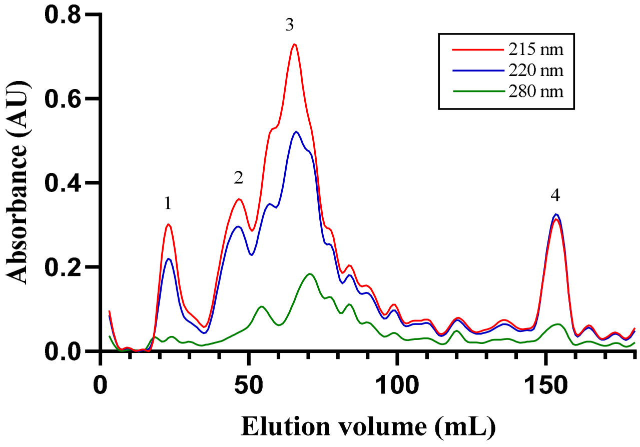

Figure S2. Elution diagram of the Sephadex G-15 column. Elution was monitored at wavelengths of 215, 220, and 280 nm. The peaks are numbered P1-P4.

A

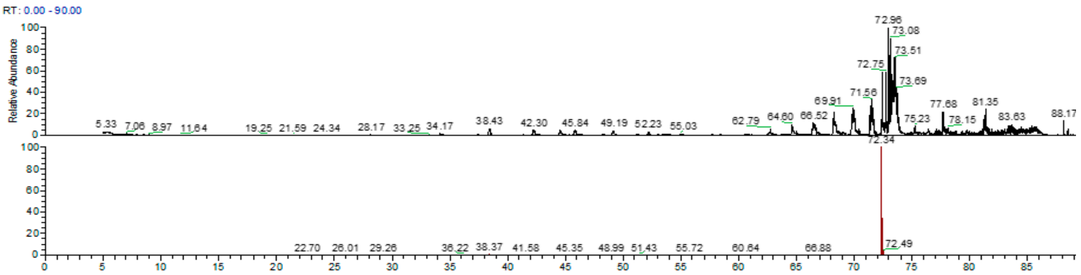

B

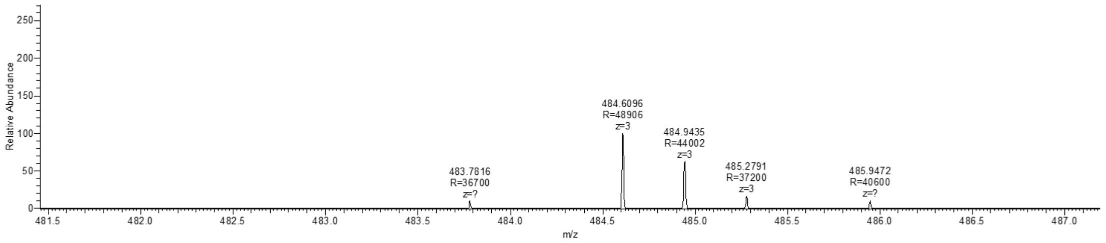

C

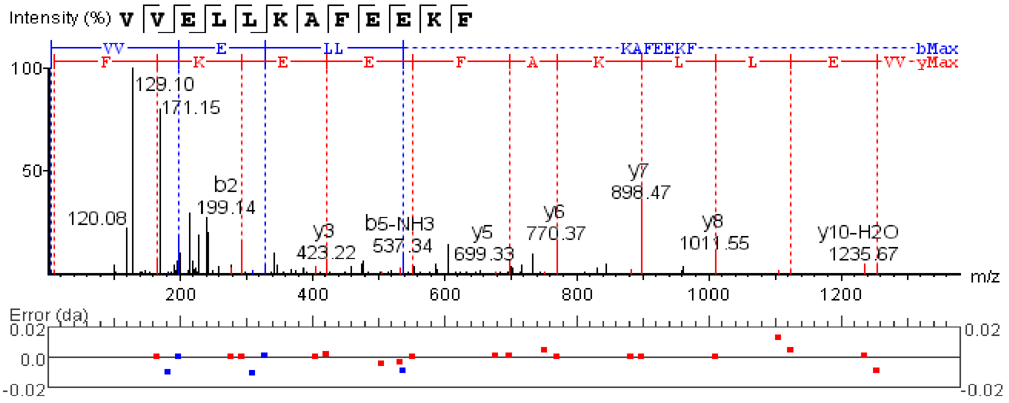

Figure S3. A: Identification of HEP (VVELLKAFEEKF) by UPLC-Q-TOF; B: MS/MS mass spectrum of dodecapeptide HEP.
